# Neutralisation of Peritoneal IL-17A Markedly Improves the Prognosis of Severe Septic Mice by Decreasing Neutrophil Infiltration and Proinflammatory Cytokines

**DOI:** 10.1371/journal.pone.0046506

**Published:** 2012-10-02

**Authors:** Jinbao Li, Yan Zhang, Jingsheng Lou, Jiali Zhu, Miaoxia He, Xiaoming Deng, Zailong Cai

**Affiliations:** 1 Department of Anesthesiology, Changhai Hospital, and Department of Biochemistry and Molecular Biology, Second Military Medical University, Shanghai, China; 2 Department of Pathology, Changhai Hospital, Second Military Medical University, Shanghai, China; 3 Department of Anesthesiology, General Hospital of PLA, Beijing, China; French National Centre for Scientific Research, France

## Abstract

**Purpose:**

The current study aimed to elucidate the role of peritoneal fluid IL-17A in septic mice, and the effects of intraperitoneal or intravenous blockade of the IL-17A pathway by anti-IL17A antibody on survival, plasma, and peritoneal cavity cytokine profile in a murine caecal ligation and puncture (CLP) sepsis model. The main source of peritoneal fluid IL-17A in septic mice was identified.

**Methods:**

Male C57BL/6 mice that underwent severe CLP or sham surgery were intraperitoneally or intravenously administered anti-IL17A antibodies or isotype antibodies. The survival rates were observed. IL-17A, TNF-α, and IL-6 cytokine levels were measured by ELISA. Surface and intracellular IL-17A immunofluorescence stains were detected by flow cytometry to identify the IL-17A–producing cells.

**Results:**

The IL-17A level was elevated much higher and earlier in peritoneal fluid than in the blood of the CLP mice. The intraperitoneal IL-17A blockade more significantly protects against CLP-induced mortality than intravenous blockade because of decreased TNF-α and IL-6 levels both in peritoneal fluid and blood, neutrophil infiltration in the peritoneal cavity, and lung injury. γδ T lymphocytes were identified to be the main source of IL-17A in the peritoneal fluid of septic mice.

**Conclusions:**

The earlier and higher elevated IL-17A derived from γδ T cells in peritoneal fluid plays a critical role during polymicrobial severe sepsis and effect of intraperitoneal IL-17A antibody administration superior to intravenous administration on survival of severe CLP-induced septic mice. The intraperitoneal blockade of IL-17A decreases proinflammatory cytokine production, neutrophil infiltration, and lung injury, thereby improving septic mice survival, which provides a new potential therapy target for sepsis.

## Introduction

Sepsis is a clinical syndrome resulting from the systemic inflammatory response to a variety of bacterial infections. Sepsis remains a prevalent clinical challenge and the underlying pathophysiology is still poorly understood. The high rates of morbidity and mortality of sepsis may result from a failure of initial pathogenic clearance or susceptibility to a secondary infection, both of which result in end-organ tissue damage. Numerous studies have demonstrated that an aberrant innate immune response early in sepsis leads to end-organ damage [1], [2]. The onset of the adaptive response to bacterial infection is thought to occur after the innate response has subsided and to maintain surveillance as well as prevent new infections [3], [4]. However, a recently emerging view points out that adaptive immune responses may be engaged much earlier in sepsis than previously thought [5]. Acute lung injury (ALI) is a common complication of sepsis. Sepsis-induced ALI is thought to be polymorphonuclear neutrophil dependent, which result in a cytokine/chemokine storm in the lungs that leads to intense ALI and acute respiratory distress syndrome [6]. Sepsis starts as a process of system inflammation mediated by pro-inflammatory cytokines/chemokines including TNF-α, IL-1, IL-6, MIP-1α, MCP-1, IFN-γ, and IL-17 as well as anti-inflammatory cytokines, e.g. IL-10 [7]. These proinflammatory cytokines result in recruitment and activation of neutrophils, NK cells, and monocytes/macrophages which produce deleterious reactive oxygen species and lysosomal enzymes [8].

The discovery of the IL-17 cytokine family has provided a new pathway for crosstalk between adaptive and innate immunity. The IL-17 family of cytokines includes IL-17A (also called IL-17), IL-17B, IL-17C, IL-17D, IL-17E (also called IL-25), and IL-17F [9]. IL-17A, the first identified member of the IL-17 family, is a proinflammatory cytokine that induces other proinflammatory mediators such as IL-6 and so on. Several sources of IL-17A have been identified, including Th17 cells, CD8+ T cells, natural killer (NK) cells, αβ T cells, γδ T cells, and neutrophils. IL-17A involvement in host defence against bacterial infection has been shown. IL-17A induces the infiltration of neutrophilic granulocytes at the infection site, as well as the expression of proinflammatory mediators such as IL-6, CXC chemokines, and matrix metalloproteinases [9]–[11]. Studies on IL-17A in sepsis has centred on elucidating its role using caecal ligation and puncture (CLP), the most frequently used and clinically relevant model for investigating the complex molecular mechanisms of sepsis. The CLP model is a peritonitis model with the clinical features of a polymicrobial infection comparable with those of peritonitis in humans. In the CLP model, sepsis originates from a polymicrobial infectious focus within the peritoneal cavity, followed by bacterial translocation into the blood compartment, which then triggers a systemic inflammatory response [12]. However, during CLP sepsis, the level and role of IL-17A within the peritoneal fluid is unclear.

Given the important role of high-level proinflammatory cytokines within the peritoneal fluid during sepsis [13], we hypothesise that elevated IL-17A in the peritoneal fluid during the early phase of severe sepsis triggers a robust and sustained systemic inflammatory response that can result in organ tissue injury and death. We measured the IL-17A levels in peritoneal fluid and compared the effects of the intraperitoneal and intravenous blockade of the IL-17A pathway using anti-IL17A antibodies. Our purpose was to elucidate the effect of intraperitoneal IL-17A neutralisation on the survival, systemic bacteraemia, and plasma proinflammatory cytokine profile of early severe sepsis in mice.

## Materials and Methods

### Severe CLP Surgery Induced Sepsis Model

All experiments were approved by the Institutional Animal Care and Use Committee of the Second Military Medical University. Adult (22 g to 30 g) C57BL/6 male mice between 8 and 10 weeks old were purchased from the Animals Experimentation Centre of the Second Military Medical University. Severe CLP-Surgery induced polymicrobial sepsis was performed as previously described [14]. Briefly, mice were anesthetized with isofluorane and a midline abdominal incision was made. The cecum was mobilized, ligated below the ileocecal valve, punctured twice with a 22 gauge needle, and a little stool was squeeze out of the cecum to induce polymicrobial peritonitis. The abdominal wall was closed in two layers. Sham-operated mice underwent the same procedure, including opening the peritoneum and exposing the bowel, but without ligation and needle perforation of the cecum. After surgery, the mice were injected with 1 mL physiologic saline solution for fluid resuscitation. All mice had unlimited access to food and water both pre- and postoperatively.

### Determination of IL-17A, IL-17F and IL-17AF Levels in Blood and Peritoneal Lavage Fluid

Plasma and peritoneal lavage fluid were harvested from mice 3, 6, 12, 24 h after undergoing severe CLP and sham surgery. The concentrations of IL-17A, IL-17F and IL-17AF were measured using murine ELISA kits (eBiosciences, San Jose, CA, USA) according to the manufacturer’s instructions. The results are expressed as picograms per millilitre of plasma or peritoneal exudates.

### Effect of Intraperitoneal or Intravenous Blockade of the IL-17A Pathway by anti-IL17A Antibody on Survival

To assess the potential therapeutic effect of IL-17A neutralisation, mice that underwent severe CLP were subsequently randomised to receive intraperitoneal or intravenous anti-IL-17A antibody (50 µg/mouse), isotype control antibody (50 µg/mouse), or PBS 3 h after severe CLP surgery. Survival was examined over the subsequent 8 days. All mice were subcutaneously administered with 1 mL of PBS after severe CLP and allowed free access to food and water. The anti-IL-17A antibody (clone: eBioMM17F3) and isotype control antibody (clone: P3.6.2.8.1) were purchased from eBiosciences (San Jose, CA, USA).

### Proinflammatory Cytokines after IL-17A Neutralisation in Severe CLP Mice

To determine the levels of circulating proinflammatory cytokines after IL-17A neutralisation, plasma and peritoneal lavage fluid were collected 12 h after anti-IL-17A or isotype antibodies were injected intraperitoneally or intravenously. Plasma was isolated from cardiopuncture-blood after anaesthesia, and peritoneal fluid was lavaged from the peritoneal cavity using 5 mL of PBS. The concentrations of TNF-α and IL-6 were measured using murine ELISA kits (Quantikine ELISA, R&D Systems, Minneapolis, MN, USA) according to the manufacturer’s instructions. The results are expressed as pg/mL of peritoneal exudates or blood.

### Lung Wet/dry Weight Ratio, Histopathologic Observation, Neutrophil Granulocyte Infiltration and Proinflammatory Cytokines

The lung wet/dry weight ratio was used to determine the different levels of lung injury after anti-IL-17A, isotype control, or PBS treatment of severe CLP mice. All mice underwent whole left lung pneumonectomy 24 h after surgery. The remaining lungs were packaged in aluminium foil, weighed, and oven dried at 70°C for 72 h to obtain pulmonary wet/dry ratios.

For histopathologic observation, specimens of the right lung were harvested and flushed with PBS, fixed with 10% formalin for 24 h, and embedded in paraffin. The sections (4 µm) were stained with haematoxylin and eosin (HE staining) for light microscopy. The total surface of the slides was scored by two blinded pathologists with expertise in assessing lung tissues. Briefly, the criteria for scoring lung inflammation were as follows [15]: 0, normal tissue; 1, minimal inflammatory change; 2, no obvious damage to the lung architecture; 3, thickening of the alveolar septae; 4, formation of nodules or areas of pneumonitis that distorted the normal architecture; and 5, total obliteration of the field. The slides were examined by two pathologists who were unaware of the groups.

Neutrophil granulocytes infiltration in lung was evaluated by detection of Gr-1 positive cells in bronchoalveolar lavage fluid (BALF). The BALF was harvested after injecting 1.5 mL of PBS totally into the lung. The neutrophil numbers were counted after centrifugation and erythrocyte lysis. Cells were also stained with fluorochrome-conjugated antibodies against cell subset–specific surface markers (Gr-1 for neutrophils). Cell numbers were calculated by obtaining the percentage by FACS analysis.

Proinflammatory cytokines IL-6 and TNF-α in BALF were determined by murine ELISA kits (Quantikine ELISA, R&D Systems, Minneapolis, MN, USA) according to the manufacturer’s instructions. The results are expressed as pg/mL.

### Mice Bacterial Clearance after Treated with Anti-IL17A Antibody

For bacterial clearance, blood and peritoneal lavage fluid samples were collected 24-h after surgery and treatment. Blood was collected by cardiopuncture after isoflurane anaesthesia administration. Peritoneal lavage fluid was harvested after injecting 2 mL of PBS into the peritoneum and serial dilution in samples (10-, 100-, or 1,000-fold in 500 µL of PBS). A 100 µL aliquot of each dilution was spread on a tryptic soy agar blood agar plate. All plates were incubated at 37°C for 24 h. Colonies were counted and expressed as colony forming units per millilitre for all samples.

### Antibodies for Flow Cytometric Analysis and Other Reagents

The antibodies for flow cytometry, purchased from eBiosciences (San Jose, CA, USA), were CD8a-PE/Cy5 (clone: 53–6.7), CD19-PE/Cy5 (clone: eBio1D3), Gr-1-FITC (clone: RB6-8C5), NK1.1-APC (clone: PK136), gamma delta TCR-FITC (clone: eBioGL3), and IL-17A-PE (clone: eBio17B7). Those that were purchased from Biolegend (San Diego, CA, USA) were CD3-PE/Cy7 (clone: 17A2) and CD4-APC (clone: GK1.5). For the detection of the major IL-17A–producing cell subsets after severe CLP, PMA (Sigma-Aldrich, St. Louis, MO, USA), ionomycin (Sigma-Aldrich, St. Louis, MO, USA), and Brefeldin A (Enzo Life Sciences, Plymouth, PA, USA) were used for intracellular IL-17A staining.

### Detection of Intracellular IL-17A in Peritoneal Lavage Fluid and Blood

To determine the major IL-17A-producing cell subset after severe CLP, peritoneal lavage fluid and blood from mice that underwent sham or severe CLP surgery were harvested 3 h later. Peritoneal cells were harvested by lavage of the peritoneal cavity with 2 mL of PBS. Whole blood (1 mL) was obtained by cardiac puncture and was drawn into tubes containing EDTA K2. Peripheral blood mononuclear cells (PBMCs) were isolated using lymphocyte separation medium (PAA Laboratories, Morningside, QLD, Austria). The peritoneal cavity cells and PBMC were centrifuged (300 *g* at 4°C for 5 min), and resuspended in fresh RPMI 1640 containing 10% foetal calf serum. The cells were then stimulated with PMA (50 ng/mL), ionomycin (500 ng/mL), and Brefeldin A (3 µg/mL) for 4 h to 6 h (37°C, 5% CO_2_, 95% humidity) as previously described [15]. The cells were first stained for the surface markers of CD3, CD4, CD8, NK1.1, Gr-1, as well as γδ T, and then with Cytofix/Cytoperm according to the manufacturer’s instructions. Intracellular cytokine staining was performed using PE-conjugated anti-IL-17A monoclonal antibodies. The stained cells were detected with a flow cytometer MACSQuant Analyzer (Miltenyi Biotec, Germany). All flow cytometry data were analysed using FlowJo ver 7.6 (Tree Star, Ashland, OR, USA).

### Determination of Neutrophil Counts in Peritoneal Lavage Fluid

Mice that underwent severe CLP were randomised to receive anti-IL-17A antibodies (50 µg/mouse), isotype control antibodies (50 µg/mouse), or PBS intraperitoneally after severe CLP surgery. The peritoneal lavage fluids of the septic and sham-operated mice were harvested 24 h after severe CLP. The neutrophil numbers were counted after erythrocyte lysis. Cells were also stained with fluorochrome-conjugated antibodies against cell subset–specific surface markers (Gr-1 for neutrophils). Cell numbers were calculated by obtaining the percentage by FACS analysis.

### Statistical Analysis

The survival of the two subgroups was estimated using a Kaplan–Meier analysis. Comparisons were performed by the log-rank test. All comparisons among groups were performed using a Mann–Whitney analysis of variance. For multigroup analysis, intergroup comparisons were performed via a Dunn’s test or Newman–Keuls multiple comparison test. All statistical analyses were performed using Prism 5.0 (GraphPad Software, San Diego, CA, USA).

## Results

### Earlier and Higher Elevated IL-17A in Severe CLP Mice Especially in Peritoneal Fluid

**Figure 1:**
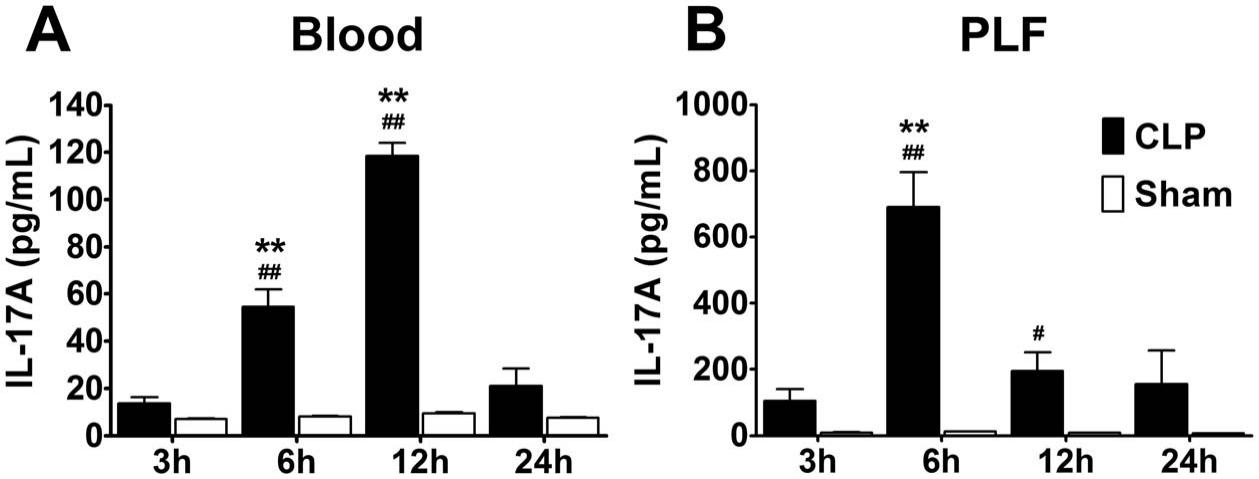
IL-17A concentration in blood and peritoneal lavage fluid after severe CLP. (A) IL-17A concentration in blood 3, 6, 12, and 24 h after the sham operation and CLP, which peaked at 12 h. (B) IL-17A concentration in peritoneal lavage fluid 3, 6, 12, and 24 h after the sham operation and CLP, which peaked at 6 h. **P<0.01: Compared with CLP 3 h group, IL-17A concentration elevated significantly. ##P<0.01 and #P<0.05: IL-17A concentration elevated significantly in CLP mice than sham group mice on the same time point.

To explore the potential role of IL-17A in sepsis, we employed severe CLP models and found elevated IL-17A concentrations in blood in CLP mice 3 h after severe CLP surgery. The peak IL-17A concentration appeared at 12 h, and a rapid decline to the baseline values occurred at 24 h (Figure 1A). However, in peritoneal lavage fluid from all CLP mice, the IL-17A level markedly increased 6 h after CLP compared with the sham mice, earlier than the time at which IL-17A was elevated in the blood. The IL-17A levels declined to baseline values 12 h after CLP (Figure 1B). The earlier and higher elevated IL-17A concentrations in peritoneal fluid after CLP indicates that localised IL-17A levels increased more and earlier than that of systemic IL-17A levels. It is worth noting that levels of IL-17F and IL-17AF change in the way of IL-17A in this setting (Data not shown). To compare the effect of intraperitoneal vs intravenous IL-17A antibody administration on survival of severe CLP-induced septic mice, neutralizing IL-17A antibody experiments were concentrated.

### Superior Effect of Intraperitoneal vs Intravenous IL-17A Antibody Administration on Survival of Severe CLP-induced Septic Mice

**Figure 2:**
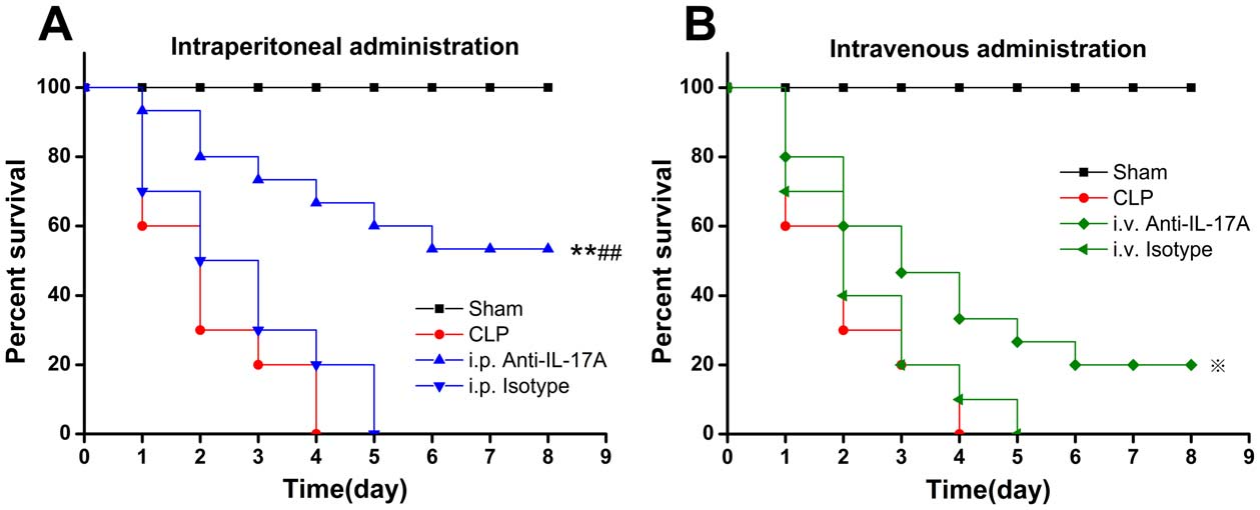
Superior effect of intraperitoneal vs intravenous IL-17A antibody administration. Intraperitoneal (A) or intravenous (B) anti-IL-17A antibody administration protected the mice from severe CLP. The CLP mice were intraperitoneally or intravenously given 50 µg of anti-IL-17A antibodies (*n* = 15), 50 µg of isotype control antibodies (*n* = 12), or 0.2 mL of PBS 3 h after severe CLP surgery. **P<0.01: i.p. anti-IL-17A compared with the CLP group; ^##^ *P*<0.01: i.p. anti-IL-17A compared with the i.p. isotype group; 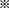 *P*<0.05: i.v. anti-IL-17A compared with the CLP group.

Mice treated intraperitoneally with anti-IL-17A antibodies 3 h after CLP showed improved 8 day survival rates (53.3%) compared with mice treated with PBS (0%; *P*<0.01) or those treated with isotype control antibody (0%; *P*<0.01) (Figure 2A). Simultaneously, the 8-day survival rates of mice intravenously treated with anti-IL-17A antibodies 3 h after CLP showed were 20% higher compared with that of mice treated with PBS (0%; *P*<0.05), but did not show a protective effect significantly compared with those treated with the isotype control antibodies (0%; *P*>0.05) (Figure 2B). Mice intraperitoneally treated with anti-IL-17A antibodies showed higher a higher 8 day survival rate (53.3%) compared with intravenously-treated mice (20%; *P*<0.05) (Figure 2).

### Level of Proinflammatory Cytokines after the IL-17A Neutralisation

**Figure 3:**
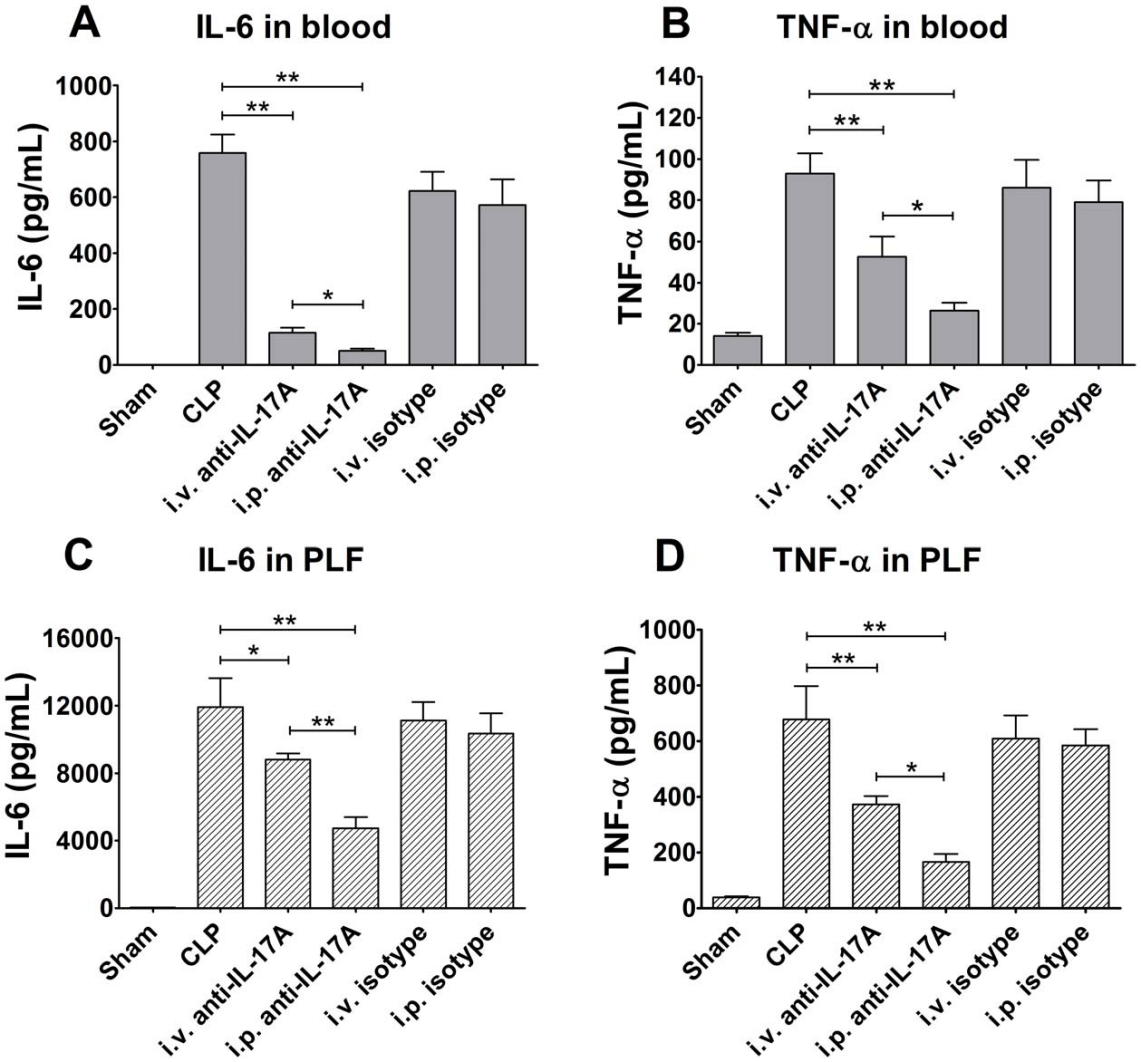
Cytokine levels in plasma and peritoneal lavage fluid after intraperitoneal or intravenous anti-IL-17A antibody blockade. The mice underwent a sham procedure, CLP, or CLP with intraperitoneal or intravenous anti-IL-17A administration (*n* = 5 for each group). The levels of TNF-α and IL-6 in the blood (A, B) and peritoneal lavage fluid (C, D) were measured 12 h after surgery. **P<0.01, *P<0.05.

To investigate the underlying potential mechanism of the protective effect of intraperitoneal anti-IL-17A neutralisation, the levels of the representative proinflammatory cytokines TNF-α and IL-6 in the peritoneal cavity and blood of severe CLP mice were measured. Both intraperitoneal and intravenous neutralisation of IL-17A markedly decreased the TNF-α and IL-6 levels in peritoneal fluid and plasma 12 h after CLP surgery compared with CLP plus PBS mice. More importantly, the concentrations of both TNF-α and IL-6 were lower after the intraperitoneal IL-17A neutralisation than after intravenous administration. The IL-6 levels decreased more significantly after the intraperitoneal administration of anti-1L-17A (Figure 3).

### Changes in the Lung Wet/dry Ratio, Histopathology, Neutrophil Granulocytes Infiltration and Proinflammatory Cytokines in BALF after IL-17A Neutralisation

**Figure 4:**
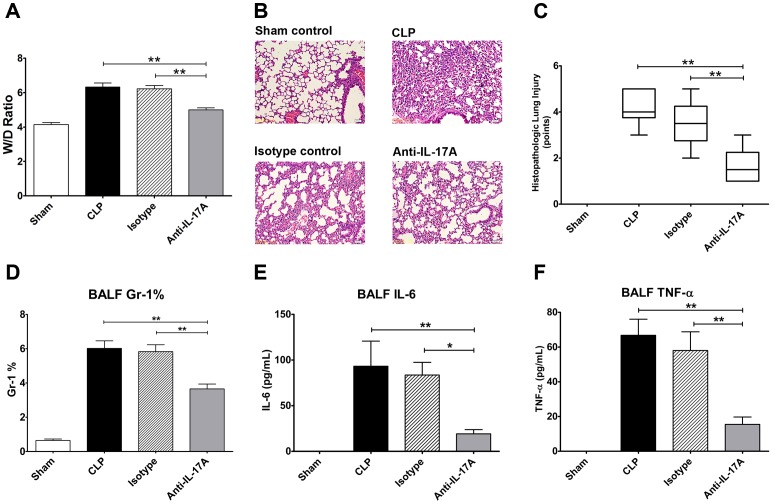
Intraperitoneal anti-IL-17A antibody administration decreased sepsis induced lung injury. The mice underwent a sham procedure, CLP, CLP with intraperitoneal anti-IL-17A, or isotype administration (*n* = 10 for each group). Bronchoalveolar lavage fluid (BALF), lung, blood, and peritoneal lavage fluid were harvested 24 h after surgery. (A) The wet-to-dry ratio was used to show lung injury in the four groups. (B) Representative HE-stained lung sections. (C) Histopathological score based on HE-stained lung sections, which showed the severity of lung injury. (D) Percentage of neutrophil granulocytes in bronchoalveolar lavage fluid (BALF) after adminitration of anti-IL-17A in the four groups. After treated with anti-IL-17A antibody after CLP and sham surgery, BALF IL-6 level (E) and TNF-α level (F) were detected to evaluate the lung injury. **P<0.01, *P<0.05.

The lungs were histologically analysed and the wet/dry weights were measured to determine lung oedema, which can partly indicate the effects of intraperitoneal anti-IL-17A antibody treatment. The wet/dry ratio of the lungs in the anti-IL-17A antibody administration mice was significantly lower than that in the CLP group or isotype-treated animals (*P*<0.01, respectively) (Figure 4A). The morphologic study showed that after 24 h of anti-IL-17A administration, the lungs of mice were damaged in the PBS-treated group. Severe oedema, wider interalveolar septa, severe alveolar haemorrhage, and extensive inflammatory cell infiltration were observed. Moderate lung oedema, haemorrhage, and inflammatory cell infiltration were seen in the intraperitoneal anti-IL-17A antibody treatment group, which suggests that severe CLP-induced lung injury decreased after IL-17A neutralisation (Figure 4B). Histopathological score further showed milder impairment in the lung after anti-IL-17A antibody administration (Figure 4C). Given that IL-17A is one of the major cytokines mediating neutrophil granulocytes infiltration, the percentage of neutrophil granulocyte was also detected in BALF and it showed the cells percentage in anti-IL-17A antibody group mice significantly decreased than that in CLP and isotype group mice (Figure 4D). Proinflammatory cytokines IL-6 (Figure 4E) and TNF-α (Figure 4F) in BALF also decreased after blockade of IL-17A intraperitoneally, indicated that anti-IL-17A antibody treatment suppressed proinflammatory response in sepsis induced lung injury.

### Bacterial Clearance after Anti-IL-17A Antibody Administration Intraperitoneally

**Figure 5:**
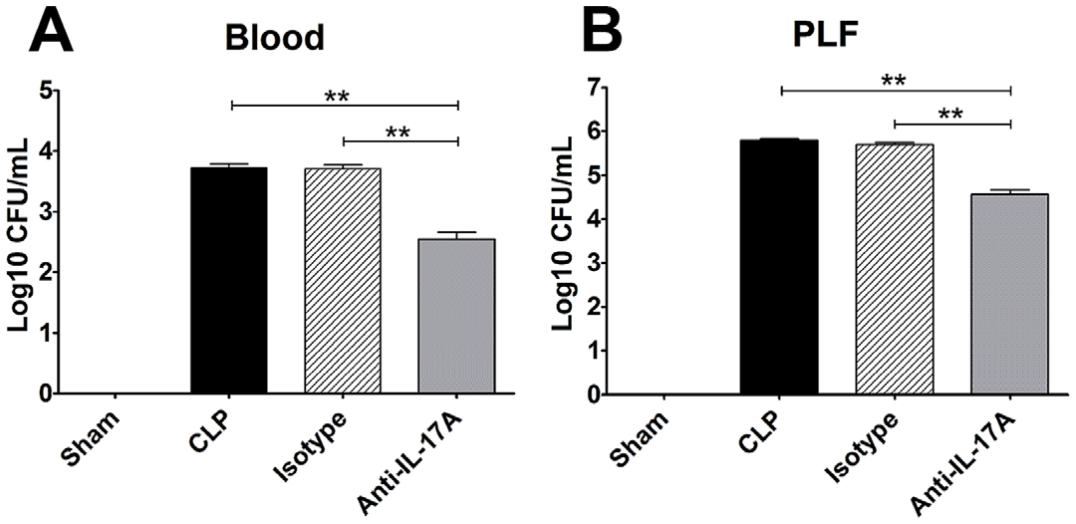
Bacterial clearance after intraperitoneal anti-IL-17A antibody administration. The mice underwent a sham procedure, CLP, CLP with intraperitoneal anti-IL-17A, or isotype administration (n = 10 for each group). Blood and peritoneal lavage fluid were harvested 24 h after surgery. Mice that received intraperitoneal anti-IL-17A antibodies exhibited a deceased bacterial burden in the blood (A) and peritoneal cavity (B) compared with mice that received isotype antibodies or PBS. **P<0.01.

Mice that intraperitoneally received anti-IL-17A antibodies showed a deceased bacterial burden in both the blood and peritoneal lavage fluid compared with those treated with the isotype antibodies or PBS (*P*<0.01, respectively) (Figures 5A and 5B).

### γδ T Lymphocytes are the Major Source of IL-17A Following CLP and Neutrophil Cell Count in Peritoneal Fluid Decreased after Intraperitoneal IL-17A Neutralisation

**Figure 6:**
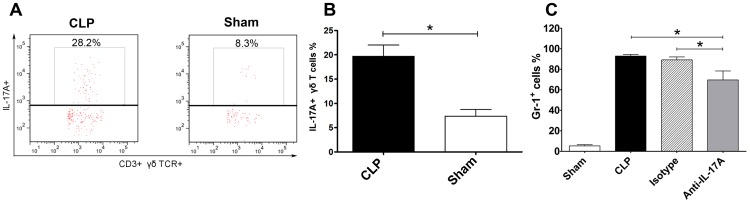
Flow cytometric analysis of IL-17A–producing cells in peritoneal fluid after CLP and the percentage of neutrophil granulocytes in peritoneal fluid after intraperitoneal blockade with anti-IL-17A antibodies. (A) Representative flow cytometric dot plots. (B) Percentage of IL-17A+ <gamma><delta>-T cells in peritoneal lavage fluid after CLP or sham operation. (C) Percentage of neutrophil granulocytes in peritoneal fluid after blockade of anti-IL-17A in the four groups. The mice underwent a sham procedure, CLP, CLP with intraperitoneal anti-IL-17A or isotype administration (*n* = 6 for each group). Peritoneal lavage fluid was harvested 24 h after surgery. *P<0.05.

We next identified the cells that produced the IL-17A in the peritoneal cavity following severe CLP surgery. About 3 h after CLP, we used an intracellular IL-17A staining, surface markers for the different cell subgroups (including CD4, CD8, CD19, NK1.1, Gr-1 and CD3), and the γδ TCR cell assay were used to detect IL-17A–producing cells. FACS analysis showed that IL-17A was mainly secreted from recruited γδ T lymphocytes (19.68% IL-17–positive γδ T cells), but not from CD4^+^, CD8^+^ T cells, CD19^+^ B cells, Gr-1^+^ neutrophils, or NK1.1^+^ NK cells (data not shown) in the peritoneal cavity (Fig. 6 A, B). These data demonstrate that γδ T cells, rather than T cells, neutrophils, NK cells, or B cells, are the major source of IL-17A production in the abdominal cavity after CLP. The neutrophil cell count expectedly decreased after intraperitoneal neutralisation in the IL-17A group compared with the CLP plus PBS or isotype antibody control groups (P<0.05) (Figure 6C).

## Discussion

The IL-17 family is emerging as an important regulator of the inflammatory response. These proteins have become the keys to a deeper understanding of the cytokine networks that coordinate and interlace innate and adaptive immunity [16]. Definitely, IL-17A is secreted by Th17 cells, CD8^+^ T cells, NK cells, αβ T cells, γδ T cells, and neutrophils. Among the numerous cells that respond to septic insult, γδ T cells are one of the key inflammatory regulators and the first line of defence against microbial invasion [17]. γδ T cells are present only in small numbers in the peripheral blood and lymphoid tissue, but are relatively abundant in tissues permanently in contact with external pathogens, namely, the skin, intestines, respiratory tract, and genital tract [18].

In the current study, we found that the IL-17A as well as IL-17F and IL-17AF levels increased significantly in both the peritoneal lavage fluid and blood in CLP mice. To compare the effect of intraperitoneal vs intravenous IL-17A antibody administration on survival of severe CLP-induced septic mice, neutralizing IL-17A antibody experiments were concentrated. The IL-17A levels peaked in the peritoneal lavage fluid earlier than in blood in all CLP mice, indicating that the level of localised IL-17A elevated earlier than that of systemic IL-17A. At the same time, we identified γδ T cells as the major source of IL-17A production in the abdominal cavity after CLP as previously described in blood [19]. Although less well characterized, IL-17A-producing γδT-cells likely require RORγt signaling IL-21 and IL-6 in their development and differentiation [20], [21]. Also, there are reports that rapid IL-17 production by γδ T cells independent of differentiation through the previously noted Th17 cells, with these cells shown as the dominant IL-17 producers in multiple murine infection models [22] These data suggest an early role for γδ T-cell mediation in murine sepsis, the effects of which likely occur before Th17 effector cell differentiation.

However, the role of the IL-17A pathway in sepsis is controversial. In one report, IL-17R deficiency is detrimental to the murine host when a non-severe CLP model is used [19], [23]. In contrast, Flierl et al [24] reported that IL-17 neutralisation in a severe model of CLP (0% 7-day survival) is successful in decreasing mortality when the anti-IL-17A antibodies are intravenously given either at the time of or 12 h after septic injury. In these two reports, bacteraemia is significantly higher in IL-17R knockout mice 6 h after non-severe CLP [22], [23], but lower in case of severe CLP after 24 h in mice given neutralising IL-17A [24], [25] compared with wild-type (WT) or isotype-treated WT mice, respectively. Similar to IL-17A in sepsis, the role of γδ T cells in sepsis is controversial with apparently conflicting data. In two separate reports, survival is decreased in γδ T cell-deficient versus WT mice in less severe CLP models (40% to 50% 7 day WT survival) [25], [26]. In direct contrast, γδ T-cell depletion in more severe sepsis models (0%–10% 7-day WT survival) did not demonstrate significant differences in survival or improved survival compared with the WT [24]. We speculate that the cause of the discrepancies among these studies is the different severities of the models used. These data may suggest that in more severe CLP models, the elevated IL-17A derived from γδ T cells induces neutrophil recruitment and increases inflammation. Consequently, organ tissue damage and mortality are increased as the blockade of IL-17A intraperitoneally decreases neutrophil recruitment in the lungs and lowers the proinflammatory cytokine levels, as in the current study. In less severe CLP models, the increased inflammation does not result in detrimental tissue damage, but allows for a significant blunting of the bacterial load and improved survival. The CLP model in the present work is a severe sepsis model (0% 7-day WT survival ) similar to that by Flierl et al [23].

Taking our findings together, we propose that γδ T cells first migrate to the peritoneal cavity and secrete IL-17A, which is the main source of systemic IL-17A during the early stage of sepsis. The abundant IL-17A in the peritoneal cavity plays a critical role in the robust and sustained inflammatory response following severe polymicrobial peritonitis and sepsis. The intraperitoneal neutralisation of IL-17A exerts protective effects against sepsis-induced organ tissue damage and lethality, which are associated with substantially reduced levels of bacteraemia and significant reductions in systemic proinflammatory cytokines. The results also suggest another explanation for the early flush abdominal liquid of sepsis patient; bacteria and foreign substances need to be eliminated, and the high levels of inflammatory factors, including IL-17, which could penetrate the blood and cause organ damage, must be decreased.

### Conclusions

The earlier and higher elevated IL-17A derived from γδ T cells in peritoneal fluid plays a critical role during polymicrobial severe sepsis and effect of intraperitoneal IL-17A antibody administration superior to intravenous administration on survival of severe CLP-induced septic mice. The intraperitoneal blockade of IL-17A decreases proinflammatory cytokine production, neutrophil infiltration, and lung injury, thereby improving septic mice survival, which provides a new potential therapy target for sepsis.
